# A novel skin pigment separation method based on sub-block selection and local clustering

**DOI:** 10.1371/journal.pone.0332849

**Published:** 2025-10-14

**Authors:** Huanyu Yang, Junzhu Zhang, Yan Ma

**Affiliations:** 1 School of Science and Technology, Shanghai Open University, Shanghai, China; 2 College of Information, Mechanical and Electrical Engineering, Shanghai Normal University, Shanghai, China; Shibaura Institute of Technology, JAPAN

## Abstract

**Background:**

Skin pigment separation is a key task in the fields of medical aesthetics, clinical analysis, and dermatological diagnosis. Existing pigment separation methods suffer from the problem of unclear separation between melanin and hemoglobin.

**Methods:**

This study proposes a novel approach that combines a sub-block selection algorithm with local clustering for skin pigment separation. The sub-block selection algorithm sorts and filters sub-blocks based on the average pixel difference, reconstructing the input data to ensure accurate separation of melanin and hemoglobin. The local clustering method uses the Euclidean distance between sample points and their surrounding cluster centers to assign sample points to their respective clusters. A random sampling of sample points is performed from each cluster to ensure convergence of independent component analysis during the iteration process.

**Results:**

Experimental evaluations demonstrate that the proposed method achieves accuracy and precise separation in skin pigment separation. Furthermore, the average success convergence rate of this approach reaches 92%, surpassing the performance of existing methods.

## Introduction

Melanin and hemoglobin are two main factors that determine the skin color [1]. The goal of skin pigment separation [2] is to extract the distribution information of melanin and hemoglobin from skin color image. Melanin and hemoglobin can more accurately measure the variations in human skin color. Therefore, pigment detection has a wide range of applications in the fields of medical cosmetology, clinical analysis, and skin medical diagnosis, among others [3–5]. The related research results of pigment analysis based on skin image processing have been applied in various skin analysis instruments. The research on skin pigment separation originated in the 1990s [6]. It can be divided into three categories:

(1) Optical properties and hardware-based: By analyzing the skin structure and its optical properties, Dawson et al. [7] found that the logarithm of the inverse of reflectance (LIR) of the skin surface is proportional to the absorbance of the skin pigment, and further propose the theoretical model concerning skin reflectance and skin pigment content. Moreover, they defined the indices including melanin index and erythema index. Based on the work of Dawson et al., Feather et al. [8,9] developed a portable reflectometer for the quantification of cutaneous hemoglobin and melanin. Masuda et al. [10] proposed to calculate the absorption spectrum of the skin from the reflection spectrum using a spectrophotometer, and determine the concentrations of melanin and hemoglobin using a multiple regression analysis, which can accurately evaluate the changes of epidermal melanin under UV irradiation. Nkengene et al. [11] tested a hyperspectral imaging device (SpectraCam® system) which can quantify melanin, hemoglobin, and oxygen saturation and detect melanin variation.
(2) Color simulation based: Relevant studies have shown that normal skin color is distributed over a certain range. Based on the Kubelka–Munk theory, Cotton et al. [12] developed a skin color prediction model that can generate all possible colors of normal skin. They found that skin colors are mainly influenced by the amount of melanin within the epidermis and hemoglobin in the dermis. The experiments demonstrate that normal skin color value lies in a plane stretched by the axis of the two factors. By analyzing the video images of in vitro cutaneous components, Takiwa et al. [13] estimated the optical parameter values of skin with a video microscope and developed a computer simulation system, which can simulate the skin color changes according to the predicted amount of melanin and hemoglobin. Li et al. [14] proposed a facial markup simulation method based on a physical reflection model. The core idea is to decompose the image into different layers, which are transformed in appearance according to the optical characteristics of cosmetics, and simulate the color effect of different cosmetics on the human face.
(3) Color image-based: By analyzing the relationship between the amount of skin pigment and color value in the optical density domain, Tsumura et al. [15] separated the spatial distributions of melanin and hemoglobin in human skin by independent component analysis (ICA) of skin color image. After that, Tsumura et al. [16] analyzed the influence of shading on skin pigment separation and proposed a shading removal model to obtain more accurate results. Building on this, Xu et al. [17] developed an automatic skin decomposition method using a single image without relying on image databases. However, their framework retained limitations in addressing surface reflection interference. To resolve this, the DL-ICA method [18] was later proposed, leveraging differences between color channels in log space to suppress such artifacts. Given its capability to overcome critical limitations of prior works (e.g., [16,17]), the DL-ICA framework [18] is adopted as the comparative baseline.

Recent advancements harness deep learning to transcend traditional pigment separation limitations, with generative models leading this evolution. Conditional Generative Adversarial Networks (cGANs) [19] now synthesize specialized skin imagery directly from standard photographs, eliminating reliance on dedicated optical hardware—Kojima et al. [20] demonstrated this by generating UV-equivalent images that accentuate pigmentation spots from conventional color photos, enabling quantitative analysis without UV equipment. Furthermore, integrated frameworks employ models like pix2pixHD [21] to produce cross-polarized images from RGB inputs, enhancing melanin and hemoglobin visualization. Beyond image translation, end-to-end regression approaches have emerged: Jung et al. [22] utilized ResUNet++ architectures trained to solve the forward problem of light-tissue interactions, theoretically reconstructing skin images from decomposed melanin, hemoglobin, shading, and specular components without pixel-level ground truths. These methods achieve high clinical correlation while offering accelerated analysis and adaptable deployment across diverse imaging environments.

While existing skin pigment separation algorithms have achieved notable theoretical progress, their practical implementation faces limitations rooted in the FastICA framework [23]. Specifically, two critical issues hinder performance: First, within the DL-ICA framework [18], the direct application of FastICA cannot effectively quantify or suppress the influence of shadow components during separation, leading to signal contamination between melanin and hemoglobin. Second, FastICA inherently exhibits instability during iterative computations, frequently failing to converge when processing skin images with diverse illumination conditions. To address these challenges, we propose a dual optimization strategy. A sub-block selection algorithm first performs secondary filtering on input skin images by sorting and filtering sub-blocks based on the average pixel difference, effectively eliminating shadow-dominated regions to improve separation accuracy. This process is further enhanced by a feature point selection algorithm that optimizes the FastICA workflow through local clustering on whitened data, where representative features are selected according to cluster centers. By reducing data volume and enforcing algorithmic stability, these refinements collectively overcome the limitations of conventional approaches.

## Related works

### Multi-layered skin model

In anatomical terms, the human skin is divided into three layers from outer to inner: the epidermis, dermis, and subcutaneous tissue [24]. In the multi-layered skin model [18], based on the pigment distribution and optical properties of skin tissues, the stratum corneum (the outermost part of the epidermis) is separately considered as a distinct layer, resulting in the skin being analyzed as a four-layer structure. From the first layer to the fourth layer, it consists of stratum corneum, viable epidermis (ranging from the stratum basale to the stratum granulosum), dermis, and subcutaneous tissue. Although histologically the stratum corneum is part of the epidermis, it is modeled separately in this study due to its distinct optical characteristics. The stratum corneum contains no pigments, consists primarily of dead keratinized cells and surface lipids, and is in direct contact with air, which gives it reflectance properties that differ significantly from the underlying layers. This separation enables more accurate modeling of light–skin interactions, particularly in the context of pigment separation analysis. The inverse of the skin reflectance can be expressed in logarithmic form [7]:


$$L=\log\left(I_{\text{0}}/I\right)=\log(1/R_{0})$$ (1)

where *L* is the logarithmic form of the reciprocal of skin reflectance, *I*_0_ is the intensity of incident light, *I* is the intensity of reflected light, and $R_{0}$ represents the total reflectance of skin tissue. Following Dawson et al.’s multi-layer model [7], $R_{0}$ is derived as:


$$R_{0}=I/I_{0}=T_{1}^{2}T_{2}^{2}T_{3}^{2}R_{4}$$ (2)

where *T*_*i*_ is the transmittance of the *i*th layer, *R*_4_ is the reflectivity of the subcutaneous tissue. This formulation inherently captures cumulative intra-layer scattering effects (e.g., diffuse scattering within the dermis) through the bulk transmittance terms, avoiding explicit modeling of inter-layer reflections. Experimental validation in [7] confirms that this simplification maintains accuracy for pigment quantification under visible light conditions.

Then we take the log of both sides of Eq (2) and combine it with Eq (1) to obtain the linear combination of skin pigment absorbance [7]:


$$L=\left(A_{1}+A_{2}+A_{3}\right)-\log\left(R_{4}\right)$$ (3)

where *A*_*i*_ corresponds to the absorbance of the *i*th layer.

The improved Lambert-Beer law [25], which extends the classical formulation to account for light scattering in biological tissues, is adopted here to model photon propagation in multilayered skin. This law is well-suited for our study, as it bridges absorption and scattering effects in heterogeneous media like skin. According to the improved Lambert-Beer law, the absorption can be expressed as a function of the absorption coefficient $\mu_{a}$ and the mean photon path length l(λ). The absorption coefficient $\mu_{a}$ is defined as the product of the extinction coefficient σ(λ) and its color concentration *c* within the tissue, i.e., $\mu_{a}=\sigma (\lambda )\times c$. The extinction coefficient σ(λ) quantifies the pigment’s intrinsic ability to absorb and scatter light at wavelength λ; in our context, we denote $\sigma_{m}(\lambda )$ for melanin and $\sigma_{h}(\lambda )$ for hemoglobin, respectively. The term color concentration *c* refers to the effective density of pigments (e.g., melanin or hemoglobin) within the skin tissue. This formulation is particularly suitable for modeling light propagation in multilayered skin structures, as it captures both absorption and scattering effects [16,17].

The absorbance A(λ) is then given by:


$$A(\lambda )=\mu_{a}\times l(\lambda )=\sigma (\lambda )\times c\times l(\lambda )$$ (4)

where λ is the wavelength.

Although *A*_*i*_ is not equal to the absorption of the *i*-th layer, according to the pigment quantification model proposed by Dawson et al. [7], the logarithm of inverse reflectance *L* can be approximately expressed as the linear superposition of the absorbance of each skin layer. The term “other pigments” mainly refers to the color contributions from fibrous proteins and subcutaneous fat, corresponding to *A*_1_ and $\mathrm{lg}(R_{4})$ in Eq (3). These components are typically not our focus in pigment separation tasks and are collectively denoted as ζ, representing the residual difference between the actual absorbance and the simplified expression of *L*. Dawson et al. experimentally demonstrated that the contributions of *A*_1_ and $\mathrm{lg}(R_{4})$ to *L* are not significant, generally less than 10%. Hence, ζ can be reasonably ignored in our model [16,17]. We therefore approximate the skin reflectance model as:


$$L\approx A_{2}+A_{3}=\sigma_{m}(\lambda )\times c_{m}\times l_{m}(\lambda )+\sigma_{h}(\lambda )\times c_{h}\times l_{h}(\lambda )$$ (5)

where $\sigma_{m}(\lambda )$ and $\sigma_{n}(\lambda )$ are the extinction coefficients of melanin and hemoglobin, *c*_*m*_ and *c*_*n*_ are their respective concentrations, and l(λ) is the mean photon path length at wavelength λ.

### DL-ICA method

For a color image pixel (*x*, *y*), its color value $C_{i}(x,y)$ (i = *R*, *G*, *B*) can be expressed as:


$$C_{i}\left(x,y\right)=k\int F\left(x,y,\lambda \right)S_{i}\left(\lambda \right)d\lambda =k\int R_{0}E\left(x,y,\lambda \right)S_{i}\left(\lambda \right)d\lambda$$ (6)

where *k* is a constant value determined by the gain of the camera and λ is the wavelength. F(x,y,λ) is the reflected spectral radiance. E(x,y,λ) is the incident spectral radiance. *R*_0_ is the surface reflectivity. $S_{i}\left(\lambda \right)$ is the spectral response function of the camera in the *i* channel (*i*=*R*, *G*, *B*). The surface reflectivity *R*_0_ is related to the pigment concentration whose relationship can be expressed as:


$$R_{\text{0}}=\exp(-\sigma_{m}(\lambda )c_{m}(x,y)l_{m}(\lambda )-\sigma_{h}(\lambda )c_{h}(x,y)l_{h}(\lambda ))$$ (7)

where $\sigma_{m}(\lambda )$ is the extinction coefficient of melanin. $c_{m}(x,y)$ is the concentration of melanin. $l_{m}(\lambda )$ is the mean path length of photons in the epidermis layer. $\sigma_{h}(\lambda )$, $c_{h}(\lambda )$ and $l_{h}(\lambda )$ are the corresponding parameters of hemoglobin in the dermis layer, respectively.

By substituting Eq (7) into Eq (6), we can obtain the equation about the image pixel color related to pigment concentration.


$$C_{i}\left(x,y\right)=k\int \exp\left\{-\sigma_{m}\left(\lambda \right)c_{m}\left(x,y\right)l_{m}\left(\lambda \right)-\sigma_{h}\left(\lambda \right)c_{h}\left(x,y\right)l_{h}\left(\lambda \right)\right\}E\left(x,y,\lambda \right)S_{i}\left(\lambda \right)d\lambda$$ (8)

Assuming that the incident light is uniform and the spectrum of the light source does not vary with direction, the incident spectral irradiance can be expressed as:


$$E\left(x,y,\lambda \right)=p\left(x,y\right)\bar{E}\left(\lambda \right)$$ (9)

where $\bar{E}\left(\lambda \right)$ is the average incident spectral irradiance, and p(x,y) represents shadow variable induced by the shape of the skin surface. By substituting Eq (7) into Eq (6) and performing the log operation on both sides, we can obtain:


$$\log\left(C_{i}\left(x,y\right)\right)=-\sigma_{m}\left(\lambda \right)c_{m}\left(x,y\right)l_{m}\left(\lambda \right)-\sigma_{h}\left(\lambda \right)c_{h}\left(x,y\right)l_{h}\left(\lambda \right)+\log(p(x,y))+\log\left(\bar{E}\left(\lambda \right)\right)$$ (10)

We define $v_{m}\left(\lambda \right)=\sigma_{m}\left(\lambda \right)\times l_{m}\left(\lambda \right)$, $v_{h}\left(\lambda \right)=\sigma_{h}\left(\lambda \right)\times l_{h}\left(\lambda \right)$, then Eq (10) can be expressed as:


$$C_{i}^{\log}(x,y)=-v_{m}(\lambda )c_{m}(x,y)-v_{h}(\lambda )c_{h}(x,y)+p^{\log}(x,y)+{\overset{―}{E}}_{i}^{\log}$$ (11)

where superscript log denotes the log operation, and *i* (=*R*, *G*, *B*) denotes the color channel.

It should be noted that in Eq (10), the term σ(λ)×l(λ) is simplified as a single variable v(λ), where σ(λ) refers to the extinction coefficient and l(λ) is the mean photon path length. This simplification does not imply that skin reflectance is determined solely by absorption. In fact, both σ and *l* are influenced by the absorption and scattering properties of the skin. As discussed in [7], The extinction coefficient σ(λ) —denoted as $\sigma_{m}\left(\lambda \right)$ for melanin and $\sigma_{h}\left(\lambda \right)$ for hemoglobin—incorporates both absorption and scattering effects. The mean path length l(λ) is also influenced by scattering in multi-layered tissues. Therefore, v(λ) is a compound variable that reflects the combined impact of absorption and scattering.

According to Eq (11), the difference between the blue channel and red channel, green channel and red channel can be obtained:


$$\{\begin{array}{c} {}*20cC_{B-R}^{\log}=\left[v_{m}\left(R\right)-v_{m}\left(B\right)\right]c_{m}+\left[v_{h}\left(R\right)-v_{h}\left(B\right)\right]c_{h}+\left({\bar{E}}_{B}^{\log}-{\bar{E}}_{R}^{\log}\right)\mathrm{\backslash vspace}4pt\\ C_{G-R}^{\log}=\left[v_{m}\left(R\right)-v_{m}\left(G\right)\right]c_{m}+\left[v_{h}\left(R\right)-v_{h}\left(G\right)\right]c_{h}+\left({\bar{E}}_{G}^{\log}-{\bar{E}}_{R}^{\log}\right) \end{array}$$ (12)

We represent Eq (12) in vector form:


$$C=V\cdot c+\bar{E}$$ (13)

where


$$C={\left[C_{B-R}^{\log},C_{G-R}^{\log}\right]}^{T}$$ (14)

$$V=\left[\begin{array}{c} {}*20cv_{m}\left(R\right)-v_{m}\left(B\right),v_{h}\left(R\right)-v_{h}\left(B\right)\\ v_{m}\left(R\right)-v_{m}\left(G\right),v_{h}\left(R\right)-v_{h}\left(G\right) \end{array}\right]=\left[v_{m},v_{h}\right]$$ (15)

$$c=[c_{m},c_{h}{]}^{T}$$ (16)

$$\bar{E}={\left[{\bar{E}}_{B}^{\log}-{\bar{E}}_{R}^{\log},{\bar{E}}_{G}^{\log}-{\bar{E}}_{R}^{\log}\right]}^{T}$$ (17)

where *c* is a two-dimensional signal that denotes the concentration of melanin and hemoglobin. The two pigment concentrations are independent of each other. The color channel difference in the optical density space can be linearly expressed as a function of $\sigma_{m}(\lambda )$ and $\sigma_{h}(\lambda )$, corresponding to melanin and hemoglobin contributions. Therefore, the calculation of pigment concentration distribution can be regarded as a blind source separation problem. The pigment image can be synthesized after solving the coefficient matrix *V* using the ICA algorithm.

### Solving with FastICA algorithm

Prior to delineating the solving steps of FastICA, it is imperative to provide a concise overview of the fundamental concepts underlying the Independent Component Analysis (ICA) algorithm. Suppose there are *n* independent source signals represented by the vector $S={\left[s_{1},s_{2},\cdots ,s_{n}\right]}^{T}$ and *n* observed signals $X={\left[x_{1},x_{2},\cdots ,x_{n}\right]}^{T}$. *n* observed signals are a linear mixture of *n* source signals, that is,


$$x_{i}=a_{i1}s_{1}+a_{i2}s_{2}+\cdots +a_{in}s_{n}$$ (18)

Eq (18) can be expressed in vector form:


X=AS (19)

where *A* is a mixing matrix and *S* is a matrix of source signals. Here, both the source data *S* and the mixing matrix *A* are unknown. How to determine the mixing matrix *A* and the source data *S* given the observation matrix *X* is the so-called blind source separation problem.

The objective of ICA is to find a separation matrix *W* so that the source data *S* can be recovered through the separation matrix *W* and the observed signals *X*. Assume that the separated signals are expressed as $y={\left[y_{1},y_{2},\cdots ,y_{n}\right]}^{T}$. Then the separation process can be expressed as:


y=WX (20)

Since the order of source signals in the results of ICA cannot be determined, the separation matrix *W* and the mixed matrix *A* have the following relationship:


$$A=W^{-1}R\Lambda$$ (21)

where *R* is a permutation matrix and Λ is a diagonal matrix that relates the absolute quantities to relative quantities.

The FastICA algorithm [26] is one of the most widely used methods in independent component analysis (ICA). ICA problems can be solved using several alternative approaches, such as nonlinear principal component analysis [27], the Infomax algorithm [28], and the fast fixed-point iteration scheme (FastICA). Among these, FastICA is favored for its computational efficiency and implementation simplicity. Moreover, it has been successfully applied in related pigment separation studies, such as those by Xu et al. [17,18], which we follow in this work to compute the separation matrix.

1. Data preprocessing: Perform centering and whitening on the mixed signal data. Centering involves subtracting the mean of the matrix E{X}, which can be expressed as:

$$\tilde{X}=X-E\left\{X\right\}$$ (22)

After centering the data, perform whitening to remove the correlation between the data points:


$$X_{white}=\lambda^{-\frac{\text{1}}{\text{2}}}U^{T}\tilde{X}$$ (23)

where U and λ represent the eigenvectors and eigenvalues of the covariance matrix $E\left\{XX^{T}\right\}$.

2. Establish the objective function: The objective function for FastICA based on negentropy is given by:

$$J\left(w\right)={\left[E\left\{G\left(w^{T}X_{white}\right)\right\}-E\left\{G\left(v\right)\right\}\right]}^{2}$$ (24)

where w is a row vector of the separation matrix W, G(•) is a non-quadratic function, and v is a Gaussian random variable with zero mean and unit variance.

3. Solve the separation matrix W: Utilizing the Newton iteration method to compute the vector w, the iterative formula is as follows:

$$w_{k+1}=E\left\{X_{white}g\left(w_{k}^{T}X_{white}\right)\right\}-E\left\{g^{\prime }\left(w_{k}^{T}X_{white}\right)\right\}w_{k}$$ (25)

where g(•) denotes the derivative of G(•). $w_{k+1}$ and $w_{k}$ represent the iterative values of w at the (k+1)-th and k-th iterations, respectively. When the absolute difference between $w_{k+1}$ and $w_{k}$ equals zero, the vector w iteration is considered to have converged. When all vectors converge, the separation matrix W can be obtained.

The pigment coefficient matrix *V* can be derived by combining Eqs (13),(20), and (21).


$$V=\left|{\left(\lambda^{-\frac{\text{1}}{\text{2}}}U^{T}\right)}^{-1}W^{-1}R\Lambda \right|$$ (26)

where *R* represents the identity matrix and Λ denotes the normalization of the result in Eq (26) to have a unit magnitude of 1.

In the DL-ICA model and its solving process, there are two main limitations that can affect the separation performance. First, it is difficult to accurately isolate and control the influence of shadow components, which often leads to undesired mixing of pigment information during separation. Second, the FastICA algorithm used for blind source separation tends to exhibit unstable convergence behavior when the input data distribution changes, especially under varying illumination or structural noise [23]. In the following section, we address these two limitations through a sub-block selection strategy and a feature point selection method based on local clustering.

## Method

**Fig 1:**
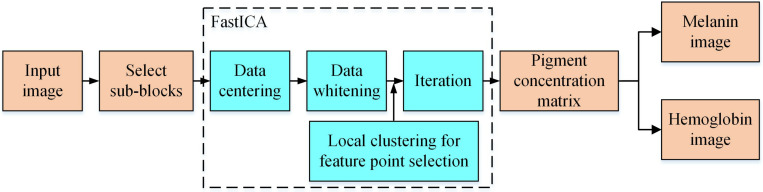
Flowchart of the proposed algorithm.

Fig 1 shows the flowchart of the skin pigment separation algorithm proposed in this paper. First, we utilize the sub-block selection algorithm to sort and filter the sub-blocks in the input image, thereby reconstructing the input data. Next, during the process of solving the separation matrix using the FastICA algorithm, we employ local clustering to select feature points. Lastly, based on the pigment concentration matrix, we derive the melanin image and hemoglobin image.

### Sub-block selection algorithm

To address the influence of shadow components, we propose a sub-block selection algorithm that filters the input image data based on local channel differences. Unlike previous methods that directly apply ICA to the entire image, our approach selects sub-blocks with more significant color channel variation, which are less affected by uniform shadows. This selection improves the signal-to-noise ratio of the input data and enhances the robustness of the separation process in the DL-ICA framework.

Prior to introducing the sub-block selection algorithm, we first analyze the actual computational errors in the skin pigment separation model.

According to Eq (9), $\bar{E}\left(\lambda \right)$ is a constant. Taking $\bar{E}\left(\lambda \right)=1$ without losing generality, we obtain:


E(x,y,λ)=p(x,y) (27)

The ideal separation result is obtained by Eq (11):


$$C_{i(ideal)}^{log}=-v_{m}(i)c_{m}-v_{h}(i)c_{h}+p^{\log}(x,y)$$ (28)

Nevertheless, there exist discrepancies between the actual computational outcomes and the theoretical values. The actual result calculated from the skin samples in the image is as follows:


$$C_{i}^{log}=-v_{mA}(i)c_{mA}-v_{hA}(i)c_{hA}+p_{A}^{\log}(x,y)$$ (29)

where $v_{mA}(i)$, $c_{mA}$, $v_{hA}(i)$, $c_{hA}$ and $p_{A}^{\log}(x,y)$ represent the actual computed values of $v_{m}(i)$, $c_{m}$, $v_{h}(i)$, $c_{h}$ and $p^{\log}(x,y)$, respectively. Let δ(x,y) be the difference between the shadow variables:


$$p_{A}^{\log}\left(x,y\right)-p^{\log}(x,y)=\delta \left(x,y\right)$$ (30)

Then Eq (29) can be expressed as


$$C_{i}^{log}=-v_{mA}\left(i\right)c_{mA}-v_{hA}\left(i\right)c_{hA}+\delta \left(x,y\right)+p^{\log}\left(x,y\right)$$ (31)

In Eq (31), we can see that if the actual result calculated by the algorithm is applied to the original image, the data δ(x,y) of the pigment part will be included in the shadow component. Therefore, when synthesizing melanin and hemoglobin images, the size of the shadow component $p_{A}^{\log}(x,y)$ is the sum of δ(x,y) and $p^{\log}(x,y)$ by default.

Next, we calculate $C_{B-R}^{\log}$:


$$C_{i}^{log}=-v_{mA}\left(i\right)c_{mA}-v_{hA}\left(i\right)c_{hA}+\delta \left(x,y\right)+p^{\log}\left(x,y\right)$$ (32)

The actual value of δ(x,y) cannot be determined during the computation process. By default, δ(x,y) and $p^{\log}(x,y)$ cancel out their corresponding values during the differencing operation. Due to the absolute value of $p_{A}^{\log}(x,y)$ being smaller than the absolute value of $C_{i}^{\log}$, the absolute value of $p_{A}^{\log}(x,y)$ may appear larger. When the values of the R, G, and B channels in the image are relatively close, the absolute values of $C_{B-R}^{\log}$ and $C_{G-R}^{\log}$ are smaller. In this case, the absolute value of $p_{A}^{\log}(x,y)$ becomes larger. δ(x,y) is then included in the background, making it difficult to accurately separate melanin and hemoglobin.

If we aim to reduce the difference between the actual value of the shadow variable $p_{A}^{log}\left(x,y\right)$ and the true value of $p^{\log}\left(x,y\right)$, we can employ the method of minimizing δ(x,y). To minimize the error between the actual results and the theoretical values, we propose a sub-block selection method. Here, we define:


$${\bar{d}}_{B-R}=mean\left(C_{B}^{\log}-C_{R}^{\log}\right)$$ (33)

$${\bar{d}}_{G-R}=mean\left(C_{G}^{\log}-C_{R}^{\log}\right)$$ (34)

$$d_{B,G-R}=\left|{\bar{d}}_{B-R}\right|+\left|{\bar{d}}_{G-R}\right|$$ (35)

where mean(·) denotes the average of the data enclosed in parentheses.

The specific steps of the sub-block selection method are as follows:

1. Divide the input image into sub-blocks of size q×q (typically ranging from 3 to 8).
2. Calculate the average difference ${\bar{d}}_{B-R}$ and ${\bar{d}}_{G-R}$ between channel values within each sub-block and compute the sum of their absolute values, denoted as $d_{B,G-R}$.
3. Sort all sub-blocks according to their $d_{B,G-R}$ values and select sub-blocks with larger $d_{B,G-R}$ values (usually the top 20% to 30%).

The size *q* of sub-blocks and the proportion of selected sub-blocks are determined based on the dimensions and characteristics of the input image. The sub-block size *q* controls the granularity of local feature extraction: smaller values of *q* capture finer color variations but may lose structural texture, while larger values preserve overall texture but may dilute local pigment features. Similarly, selecting too few sub-blocks may result in inadequate data for separation, whereas selecting too many may reintroduce the effects of shadow or noise, making the separation similar to that of unfiltered input. Based on empirical evaluation, we typically set *q* within the range of 3–8, and retain 20% to 30% of the sub-blocks with the highest feature differences, which achieves a good balance between detail retention and shadow suppression.

By employing a sub-block selection approach, each small block preserves the same tissue structure as the original skin image while preventing excessive similarity between channels.

### Feature point selection

**Fig 2:**
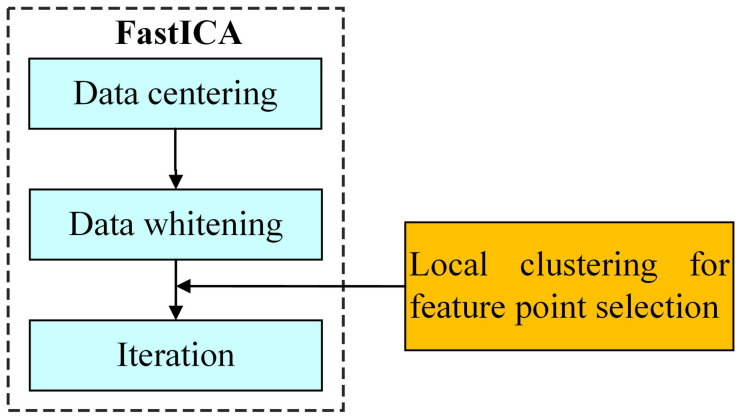
FastICA algorithm and feature point selection.

As shown in Fig 2, the FastICA algorithm consists of three steps: data centering, data whitening, and iteration. In the pigment separation algorithms, it is usually necessary to use clear original images as the data source to ensure recognizability. However, due to the instability of the FastICA iterative process, the separation matrix often fails to converge when solving the pigment separation results. Therefore, we propose a feature point selection method based on local clustering to further sparsify the data in the skin region and choose an appropriate number of sample points to optimize the iterative convergence of FastICA.

Next, we will provide a detailed explanation of the feature point selection algorithm.

Suppose that $Z=[z_{1},z_{2},\cdots ,z_{n}{]}^{T}$ represents the whitened data and k(k≤n) represents the number of cluster centers.

1. Distribute *K* initial cluster centers $P=\left[p_{1},p_{2},\cdots p_{K}\right]$ evenly within the sample points:

$$p_{i}=\frac{\sum z_{j}}{\left\lfloor \frac{n}{k}\right\rfloor },\left(i=1,2,\cdots ,k\right)$$ (36)

where *z*_*j*_ represents the sample points within a $\sqrt{\left\lfloor \frac{n}{k}\right\rfloor }\times \sqrt{\left\lfloor \frac{n}{k}\right\rfloor }$ neighborhood range centered around *p*_i_.

**Fig 3:**
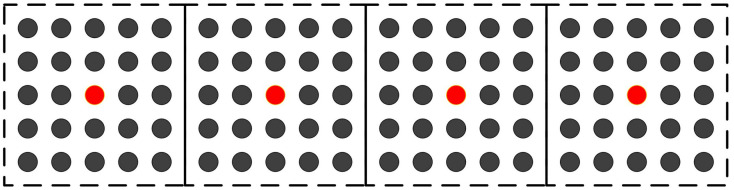
Assign initial cluster centers.

Fig 3 provides an example of uniformly distributing initial cluster centers, where n=100, k=4, and $\left\lfloor \frac{n}{k}\right\rfloor =25$. The black dots represent the sample points, and the red dots represent the initial cluster centers. In Fig 3, the dashed box represents a 5×5 neighborhood centered around *p*_*i*_.

2. Calculate the distance between the sample point and its neighboring cluster centers, and update the labels of the sample points.

Set the search range be *r*. For each sample *z*_*j*_, if it belongs to the cluster center *p*_*i*_, calculate the distance from *z*_*i*_ to the neighboring cluster centers $p_{j-r},\cdots ,p_{j-1},p_{j},p_{j+1},\cdots ,p_{j+r}$:


$$dist\left(p_{t},z_{j}\right)={\left\|p_{t}-z_{j}\right\|}_{2}$$ (37)

where t=j−r,⋯,j−1,j,j+1,⋯,j+r. Assign the cluster center *p*_*i*_ with the minimum distance to *z*_*j*_ as the cluster to which *z*_*j*_ belongs. Update the class label of *z*_*j*_ to *i*.

3. Update the cluster centers

Compute the mean value of all sample points within each cluster *c*_*i*_ and update the cluster center *p*_*i*_ with this value.


$$p_{i}=\frac{\text{1}}{\left|c_{i}\right|}\sum_{j\in c_{i}}z_{j},\left(i=1,2,\cdots ,K\right)$$ (38)

4. Iteration process

Repeat steps 2 and 3 until the class labels of the sample points no longer change between consecutive iterations. In practice, we define convergence as the condition where fewer than 1% of the sample points change their cluster assignments in an iteration. This threshold balances efficiency and stability: allowing a small margin avoids unnecessary iterations caused by minor fluctuations. Once this condition is met, the cluster centers corresponding to each point remain stable, and the clustering process is considered complete. If the convergence condition is too strict, the algorithm may require excessive computation without significantly improving accuracy; if too loose, it may lead to unstable or inaccurate feature point selection. The adopted condition was determined empirically based on experimental stability across a range of skin images.

5. Feature point selection method

To avoid under-sampling or overfitting during feature point selection, we adopt a controlled sampling strategy within each cluster. Assume that the number of basic feature points is *m*. For each cluster, we randomly select *m* representative sample points. If a cluster contains fewer than *m* samples, we retain all available points. This strategy ensures a consistent sampling density across clusters, while preventing sparse clusters from being overrepresented or dense clusters from contributing excessive redundancy.

Through empirical testing across various image sizes and clustering configurations, we found that choosing *m* in the range of 3–5 yields effective convergence behavior. Smaller values (e.g., *m* = 1 or 2) may lead to insufficient representation of cluster characteristics, while larger values (e.g., *m* > 5) tend to introduce overlapping or redundant information that negatively affects the stability of FastICA. In all clustering-based experiments, we set *m* = 3 per cluster unless otherwise specified.

This sampling scheme directly influences the input size of ICA and thus affects its convergence rate. As demonstrated in Figs 7 and 8, the local clustering approach with controlled feature selection significantly improves the success convergence rate of FastICA compared to other methods. Therefore, our method achieves a balance between computational efficiency and separation robustness by combining localized clustering with fixed-point sampling per cluster.

**Fig 4:**
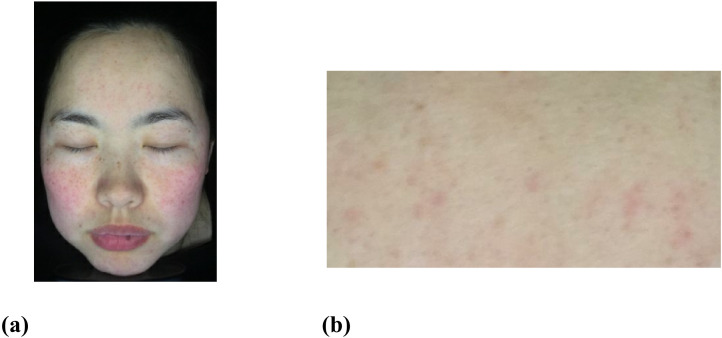
A sample image. (a) The original image. (b) Rectangular skin area on forehead. Reprinted with permission from Shanghai Siyan Software Technology Co., Ltd. under a CC BY license.

**Fig 5:**
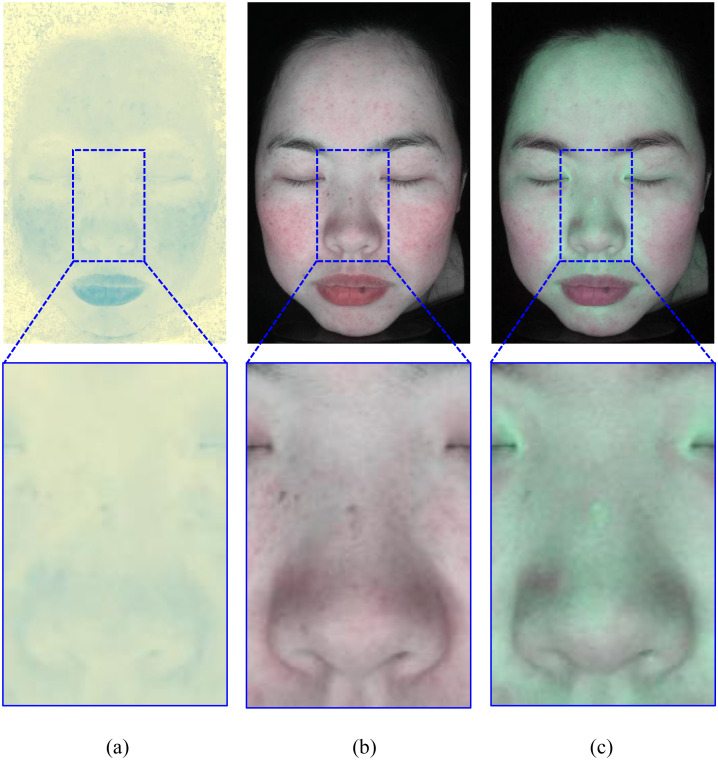
Hemoglobin images obtained from three methods. (a) Method 1 (b) Method 2 (c) Our method. Reprinted with permission from Shanghai Siyan Software Technology Co., Ltd. under a CC BY license.

**Fig 6:**
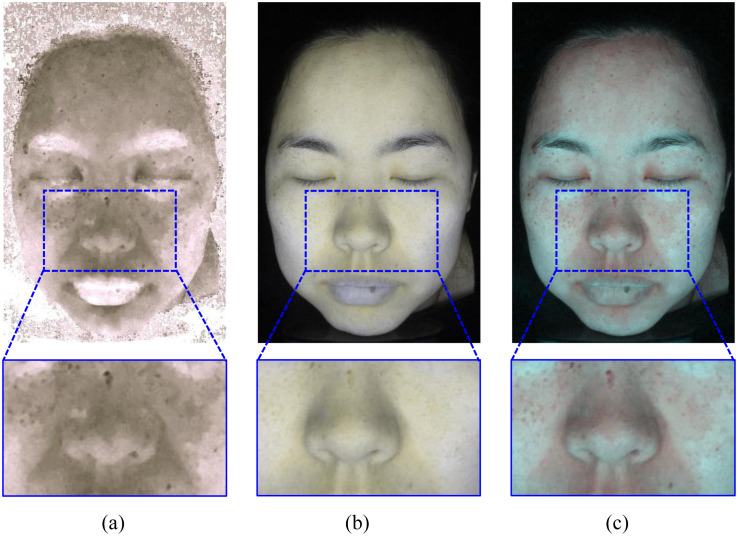
Melanin images obtained from three methods. (a) Method 1 (b) Method 2 (c) Our method. Reprinted with permission from Shanghai Siyan Software Technology Co., Ltd. under a CC BY license.

### Image synthesis

Following the data processing of the skin image and obtaining the separation matrix through FastICA algorithm, the synthesis of the pigment image is performed. Firstly, the elements in *V* are rearranged.


$$V=\left[\begin{array}{cc} {}*20cv_{11} & v_{12}\\ v_{21} & v_{22} \end{array}\right]$$ (39)

If the ratio satisfies: $\frac{v_{21}}{v_{11}}\in (1,6.33]$, $\frac{v_{22}}{v_{12}}\in [0.48,1)$, then $v_{m}=\left[\begin{array}{c} {}*20cv_{11}\\ v_{21} \end{array}\right]$, $v_{h}=\left[\begin{array}{c} {}*20cv_{12}\\ v_{22} \end{array}\right]$, otherwise $v_{m}=\left[\begin{array}{c} {}*20cv_{12}\\ v_{22} \end{array}\right]$, $v_{h}=\left[\begin{array}{c} {}*20cv_{11}\\ v_{21} \end{array}\right]$; The elements in *V* are rearranged to satisfy the condition: $V^{*}=\left[\begin{array}{cc} {}*20cv_{m} & v_{h} \end{array}\right]$.

Substitute the RGB channel values $H_{i}$ ($={\left[H_{R}\;\;\;,H_{G}\;,\;\;H_{B}\right]}^{T}$) of the original image into Eq (12), we obtain $H={\left[H_{G-R}^{\log},H_{B-R}^{\log}\right]}^{T}$. We can obtain the pigment concentration matrix *c* by combining the pigment coefficient matrix V^*^:


$$c={\left(V^{*}\right)}^{-1}\left(H-\bar{E}\right)$$ (40)

In practical computations, the value of $\bar{E}$ is commonly set to 0.

The melanin image and hemoglobin image are represented as follows:


$$L_{m}=\exp\left(V^{*}\cdot \left[\begin{array}{cc} {}*20c1 & 0\\ 0 & 0 \end{array}\right]\cdot c+\left[\begin{array}{c} {}*20cH_{R}^{\log}\\ H_{R}^{\log} \end{array}\right]\right)$$ (41)

$$L_{h}=\exp\left(V^{*}\cdot \left[\begin{array}{cc} {}*20c0 & 0\\ 0 & 1 \end{array}\right]\cdot c+\left[\begin{array}{c} {}*20cH_{R}^{\log}\\ H_{R}^{\log} \end{array}\right]\right)$$ (42)

We can obtain the values of the B and G channels for the melanin image and hemoglobin image according to Eqs (41) and(42), respectively. By combining these values with the R channel values of the original image, we obtain the melanin image and hemoglobin image of the skin.

### Ethics statement

The facial images used in this study were all original measurement images provided by Shanghai Siyan Software Technology Co., Ltd. No images from public datasets, the internet, or previously published works were used. The company conducted data collection between October 2021 and April 2022, during which written informed consent was obtained from all participants. Each participant voluntarily signed a consent form after being informed of the study’s purpose and procedures.

The facial images were captured using a professional facial imaging system based on cross-polarized light, which minimizes surface reflections and enhances the visibility of subsurface skin features such as pigmentation and vascular structures. During image acquisition, participants were seated in a controlled environment with a black background and uniform lighting to ensure consistent illumination and minimize external interference. The use of cross-polarized imaging improves the reliability of pigment separation algorithms by reducing specular reflection and enhancing contrast in melanin and hemoglobin distributions.

The individual in this manuscript has given written informed consent (as outlined in the PLOS consent form) to publish these case details. The signed consent forms are securely filed by the data provider.

Since the data were collected with full consent and no personally identifiable information is disclosed in the manuscript, and considering that the data provider ensured participant privacy and study compliance, no additional ethics committee approval was sought for this study.

## Results

### Dataset description and external validity

The dataset used in this study includes 50 facial images collected from 50 unique individuals, all of Chinese ethnicity, aged between 25 and 45 years. The gender distribution is approximately balanced, with 26 female and 24 male participants. Based on the Fitzpatrick skin type classification, all subjects fall within Type III to IV, which is representative of East Asian populations.

All facial images were collected using a high-resolution professional facial imaging system developed by Shanghai Siyan Software Technology Co., Ltd., equipped with cross-polarized lighting and standardized RGB sensors. During image acquisition, participants were seated in a controlled indoor environment with a black background and uniform diffuse lighting, ensuring consistent illumination and minimal specular reflections. Cross-polarized imaging was adopted to enhance subsurface pigment visibility while suppressing surface glare.

It should be noted that the current dataset is limited in diversity, as it represents a single ethnic group and skin type range (Fitzpatrick III–IV). Consequently, the external validity of the proposed method across other populations—such as individuals with Fitzpatrick Types I–II (lighter skin) or Types V–VI (darker skin)—has not yet been evaluated. However, as the method relies on data-driven mechanisms (e.g., sub-block selection and local clustering) rather than hard-coded skin characteristics, it is potentially adaptable to other skin types. Future work will expand the dataset to include broader ethnic and phototype diversity, and incorporate clinical validation with dermatologists to assess generalizability across clinical and demographic settings.

### Performance of sub-block selection algorithm

This section presents the evaluation of the sub-block selection algorithm. The experimental data consists of 50 face images captured under cross-polarized light conditions. The original image, as shown in Fig 4(a), has dimensions of 1501 pixels wide and 2301 pixels high. For the purpose of analysis, a rectangular region of skin area measuring 300 pixels in width and 600 pixels in height is chosen as the input for data analysis. Care has been taken to exclude non-skin areas such as eyes, nose, and eyebrows, to minimize interference from unrelated data.

In this study, a rectangular region on the forehead was manually selected as the input region for pigment separation, as it provides a relatively large, flat, and uniform skin area. Non-skin regions such as the eyes, eyebrows, and nose were manually excluded to minimize interference. The same forehead region was used consistently across all comparative methods to ensure fairness in evaluation.

The proposed method does not require a specific region shape or size, as the sub-block selection and local clustering are applied only within the selected region. During local clustering, no special treatment is applied to the boundary pixels of the selected region. This is because the algorithm assumes that human facial skin exhibits local texture similarity, and the clustering process is based on local neighborhoods rather than global continuity. Therefore, minor discontinuities at the edges do not significantly affect the clustering results or convergence performance.

In the subsequent experiments, the selected rectangular skin image from the forehead area is depicted in Fig 4(b). 20% of the sub-block pixels are chosen to recombine the input data using the sub-block selection method. A total of 7200 sub-blocks are obtained, which are then substituted into Eq (12) to calculate the corresponding data matrix *C*.

The experimental results of the shadow removal method proposed by Tsumura et al. [16] (hereinafter referred to as Method 1) and the DL-ICA method proposed by Xu et al. [18] (hereinafter referred to as Method 2), which improves upon their earlier framework [17] by incorporating the difference of each color channel in log space to eliminate surface reflection interference, are compared with the experimental results of the sub-block selection algorithm.

Fig 5 illustrates the hemoglobin images obtained from the three methods. Compared with the original image, both Method 1 and Method 2 (Fig 5(a) and 5(b)) are able to capture the general distribution of hemoglobin. However, they fail to accurately differentiate hemoglobin from melanin in certain areas. For example, melanin spots near the nasal bridge are erroneously included in the hemoglobin images generated by Method 1 and Method 2, as highlighted in the enlarged region. This indicates an insufficient separation between the two pigments. In contrast, our method (Fig 5(c)) produces a hemoglobin map that better suppresses melanin interference, particularly around the bridge of the nose, the cheeks, and the area around the eyes. The evaluation is based on visual comparison with the original image, where clearer boundaries and pigment-specific localization indicate a more accurate separation.

Fig 6 shows the melanin images obtained from the three methods. Method 1 (Fig 6(a)) can highlight melanin spots but fails to suppress shadow-induced artifacts, especially on both sides of the face and the nasal area. These regions, which appear dark due to lighting, are incorrectly interpreted as melanin. Method 2 (Fig 6(b)) provides a more comprehensive estimation of melanin distribution and corrects some shadow effects, but it lacks the spatial resolution to capture local pigment spots. Our method (Fig 6(c)) combines the advantages of both approaches. It effectively removes shadow interference while preserving detailed melanin spot information, as shown in the enlarged area. This improvement benefits from the proposed sub-block selection algorithm, which adaptively selects facial regions to enhance pigment separability while mitigating lighting artifacts.

Although all experiments in this section were conducted on the forehead region to ensure consistency and minimize the interference from occlusions and non-skin elements, the proposed method is not limited to this area. The sub-block selection and local clustering algorithms are region-independent and can be applied to other facial areas such as the eyebrow and eye. To qualitatively illustrate this, we provide additional examples in Supplementary S1 Fig showing pigment separation results when applying our method to rectangular regions on the eyebrow, eye, and nose. These regions exhibit more complex textures, curvature, and specular reflections, which can negatively impact separation quality. As shown, the pigment maps generated from these areas are less stable compared to those from typical skin regions. This supports our choice of using the forehead for performance benchmarking.

### Performance of local clustering

This section presents the experimental results of feature point selection using local clustering. Six facial images captured under cross-polarized light were used, and four methods were evaluated: the shadow removal method (Method 1), the DL-ICA method (Method 2), the Sub-block selection algorithm (Method 3), and the proposed local clustering method (our method), which is implemented based on Method 3.

In all experiments, the same facial region was used across methods. Input sizes ranged from 4,000–75,000 pixels, with a step of 500 pixels. Since FastICA involves random initialization of the separation matrix, each method was run 100 times per input size to assess its stability, and the success convergence rate was calculated accordingly.

**Fig 7:**
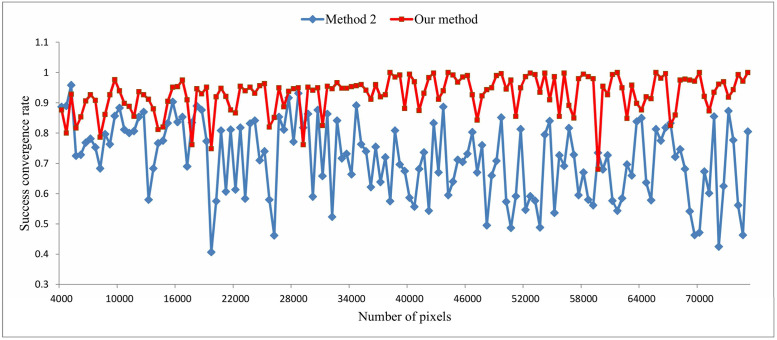
Success convergence rates of Method 2 and our method.

**Fig 8:**
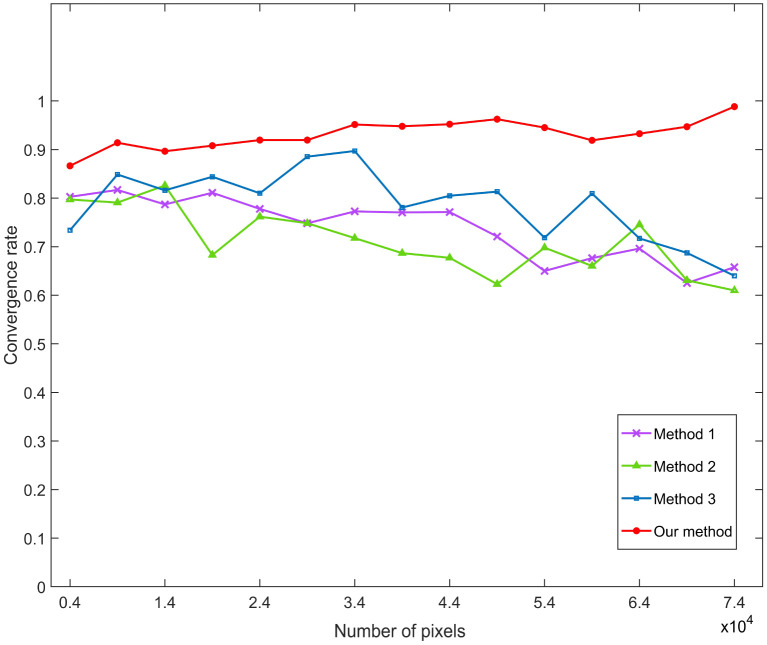
Success convergence rates of the four methods.

To emphasize the improvement achieved by our method over its base method (Method 2), Fig 7 presents a direct comparison between the two. Fig 8 provides a comprehensive comparison of all four methods, where each point represents the average convergence rate from 10 batches of experiments. The results show that our method consistently achieves higher convergence rates, especially as image size increases. A high convergence rate is crucial for real-world applications. Poor convergence can result in inconsistent pigment separation, requiring repeated parameter tuning or even re-acquisition of images, thereby increasing time and labor costs.

**Fig 9:**
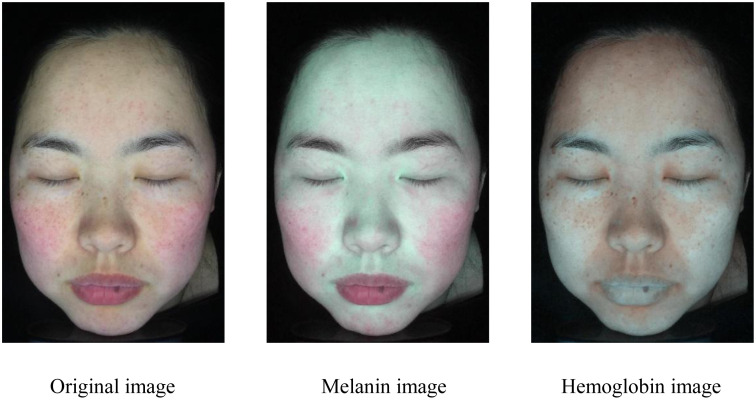
Pigment separation results of sample image 1. Reprinted with permission from Shanghai Siyan Software Technology Co., Ltd. under a CC BY license.

**Fig 10:**
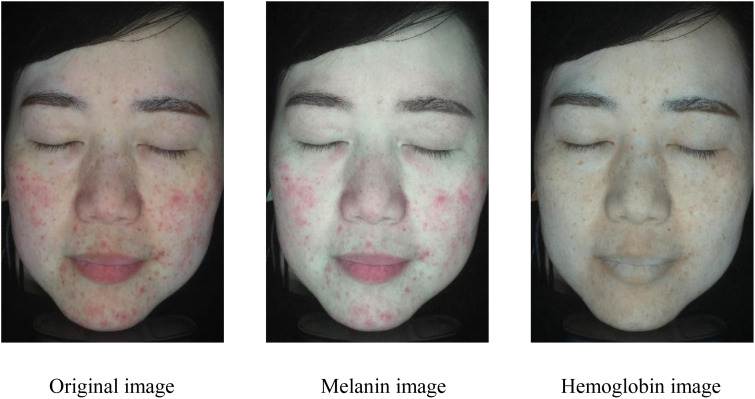
Pigment separation results of sample image 2. Reprinted with permission from Shanghai Siyan Software Technology Co., Ltd. under a CC BY license.

**Fig 11:**
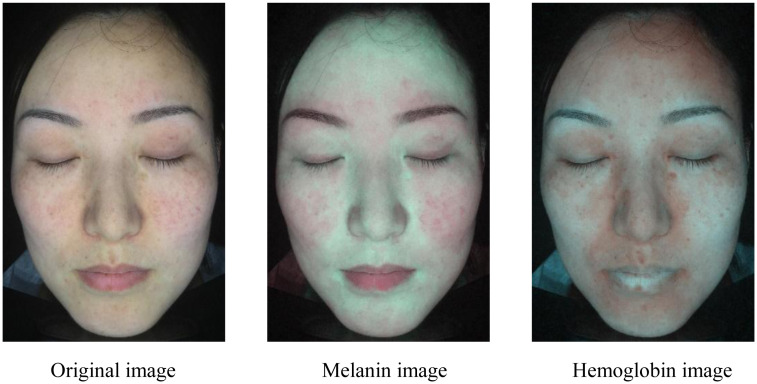
Pigment separation results of sample image 3. Reprinted with permission from Shanghai Siyan Software Technology Co., Ltd. under a CC BY license.

Table 1 presents the average success convergence rates of the four methods. Our method exhibits an average success convergence rate of 0.9276, which is higher than the other three methods. Compared to Method 3, we can observe that the local clustering method significantly improves convergence efficiency. In addition, Figs 9–11 present the results of pigment separation on three facial images using our method. It is evident from Figs 9–11 that our method effectively separates the hemoglobin and melanin in skin images. The experiments demonstrate that our method, using single-image data, exhibits higher stability in pigment separation and maintains better separation effects when processing the same fixed skin area. This advantage becomes more pronounced as the volume of image data increases.

**Table 1:** Total average convergence rate of the four methods.

Method	Method 1	Method 2	Method 3	Our method
Convergence rates	0.7451	0.7175	0.7960	0.9276

In our proposed method, certain fixed parameters are used, such as sub-block size and selection ratio (Section: Sub-block selection algorithm), as well as the convergence threshold in local clustering (Section: Feature point selection). These values were determined empirically and have a measurable impact on the convergence rate and separation quality. However, as discussed in earlier sections, the convergence condition was designed to balance efficiency and accuracy, and does not negatively affect the overall performance.

Regarding the compared methods (Method 1 and Method 2), no additional parameter tuning or manual adjustment was applied; we implemented these methods strictly according to their respective original literature [16,17]. All methods were tested using the same input region, which was manually selected from the forehead area, and all algorithms were applied to identically preprocessed data to ensure a fair comparison.

Therefore, the observed improvement in convergence rate can be attributed to the design of the proposed method, rather than to differences in data preprocessing or parameter tuning between methods.

To ensure a fair and reproducible comparison, all baseline methods were implemented in accordance with their original designs and parameter configurations:

- Method 1 (Tsumura et al. [16]): Used default settings for shading removal and ICA computation, as described in the original study.
- Method 2 (DL-ICA [18]): Followed the procedure of taking logarithmic differences between RGB channels and applying FastICA directly to the transformed data. The FastICA implementation used the standard whitening and fixed-point iteration scheme with no additional tuning.

For all methods, the same input region (forehead skin) was used, and no extra pre-processing or filtering was applied beyond what was prescribed in the original publications. We did not perform hyperparameter sweeps for the baseline methods, as they do not include tunable parameters in their canonical forms, and the goal was to replicate their reported behavior rather than optimize them under new conditions. This consistent evaluation strategy allows us to highlight the performance improvements achieved by our proposed framework, which introduces data selection and clustering mechanisms not present in prior works.

In this study, the convergence rates presented in Table 1 are based on randomly selected facial images, with each image representing a unique subject. While we ensured subject independence, we did not perform formal statistical tests such as paired t-tests, nor did we report standard deviations or confidence intervals. Our choice was motivated by the exploratory nature of this study and the limited dataset size. We acknowledge this as a limitation and plan to incorporate more rigorous statistical evaluation—including reporting mean ± SD, 95% CIs, and effect sizes—in future work.

### Parameter sensitivity analysis

In our method, three key parameters may affect the performance of pigment separation: the sub-block size (*q*), the number of clustering centers (*k*), and the percentage of selected sub-blocks used for ICA. To evaluate the sensitivity of each parameter independently, we conducted controlled experiments where only one parameter was varied at a time, while the other two were fixed to reasonable default values.

Unless otherwise specified, we set the sub-block size *q* to 5, the number of clustering centers *k* to 1200, and the selected block percentage to 25%. These values were chosen based on empirical performance and represent a good trade-off between efficiency and accuracy in our method. The following subsections detail the sensitivity analysis for each parameter individually.

**Fig 12:**
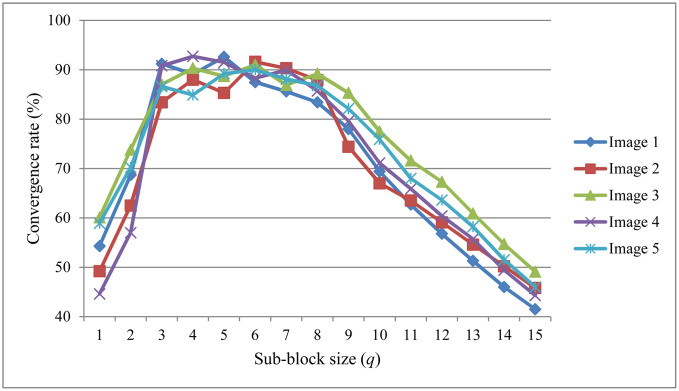
Convergence rate of FastICA under different sub-block sizes *q.*

To evaluate the sensitivity of our method to the sub-block size *q*, we conducted experiments on five cross-polarized facial images, varying *q* from 1 to 15. For each value of *q*, we measured the convergence rate of the FastICA algorithm, defined as the proportion of 10 runs that successfully completed pigment separation. As shown in Fig 12, the convergence rate increases quickly when *q* grows from 1 to 3, and stays at a relatively high level between *q* = 3 and *q* = 8 across all five images. This supports our empirical choice of using *q* within this range, which offers a good balance between capturing local texture details and keeping the overall structure intact. When the sub-block size is too small, the results become unstable due to noise. When it is too large, important local pigment features may be smoothed out, making separation less accurate.

To further evaluate the robustness of our method, we conducted a sensitivity analysis on the number of clustering centers *k*, which determines how many representative samples are used in FastICA. We varied *k* from 100 to 3000 and measured the convergence rate across five cross-polarized facial images. For each value of *k*, we ran the algorithm 10 times and calculated the proportion of runs that successfully completed the pigment separation task.

**Fig 13:**
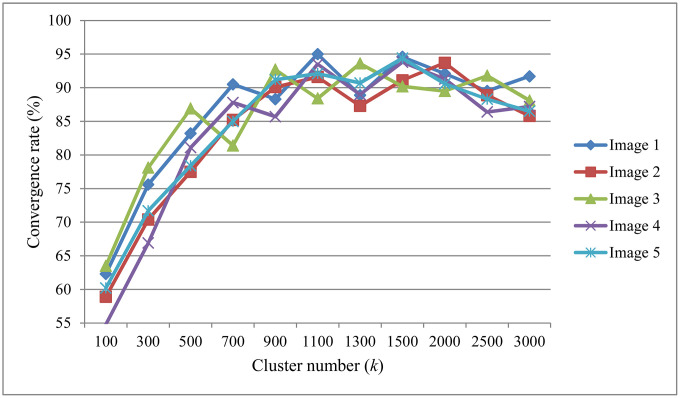
Convergence rate of FastICA under different cluster number *k.*

As shown in Fig 13, the convergence rate increases quickly as *k* grows from 100 to around 1100, suggesting that more representative samples lead to better and more stable ICA performance. However, when *k* becomes too large (above 1300), the convergence rate starts to fluctuate and even slightly drops in some cases. This may be caused by too many similar or overlapping samples being included, which can reduce the effectiveness of the separation. Overall, the best results are achieved when *k* is between 900 and 1500, where all five images show consistently high convergence rates above 90%. This supports our choice of using a moderate number of clusters to ensure both accuracy and efficiency.

**Fig 14:**
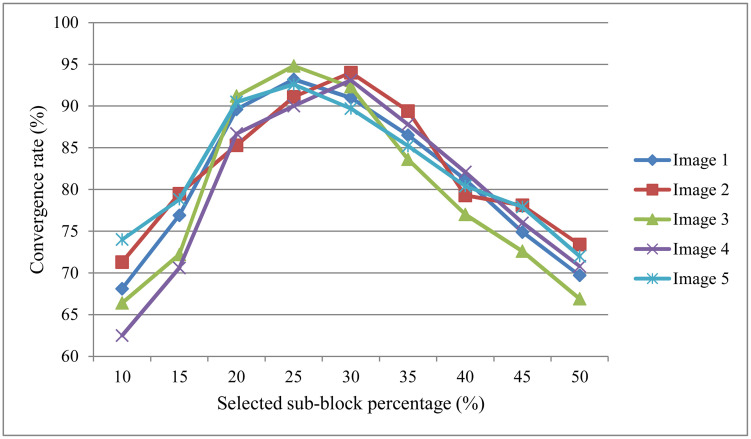
Convergence rate of FastICA with different selected sub-block percentages.

We also investigated the sensitivity of our method to the percentage of selected blocks used for ICA. After clustering, we varied the proportion of retained blocks from 10% to 50% and evaluated the convergence rate on five cross-polarized facial images. As shown in Fig 14, the convergence rate increases from 10% to 25%, reaching the highest performance between 25% and 30%. This suggests that using too few blocks may lose important structural information, while including too many may introduce redundant or noisy regions that hinder separation quality. The results support our empirical choice of selecting around 25%–30% of clustered regions, which provides a good trade-off between data compactness and separation reliability.

### Runtime analysis

To comprehensively evaluate the computational efficiency of pigment separation methods, we conducted runtime comparisons under a standardized environment using MATLAB R2019a on a PC equipped with an Intel® Core™ i7-13700 processor and 32GB RAM. Five face images of varying complexity were processed by Method 1, Method 2, and our proposed approach. The execution time for each method is summarized in Table 2.

**Table 2:** Execution time for Method 1, Method 2, and our method (second).

Method	Image 1	Image 2	Image 3	Image 4	Image 5	Average
Method 1	0.878	0.965	0.984	0.946	0.959	0.946
Method 2	0.932	0.959	0.943	0.952	0.951	0.947
Our method	0.643	0.857	0.659	0.669	0.802	0.726

Our method achieves a 23.3% reduction in average runtime (0.726 seconds), compared to Method 1 (0.946 seconds) and Method 2 (0.947 seconds). This improvement results from two algorithmic optimizations. First, the sub-block selection phase eliminates low-information regions (e.g., homogeneous or shadowed areas), reducing the initial data volume by approximately 75%. Second, the local clustering step condenses the remaining data into 1,200 representative feature points, significantly reducing the input size for FastICA—the most computationally expensive component. This dual-stage compression effectively mitigates the quadratic time complexity $O(n^{2})$ associated with FastICA, leading to faster overall execution.

## Conclusion

This study focuses on exploring skin pigment separation methods. Firstly, precise separation of skin pigments is achieved through the sub-block selection algorithm. Subsequently, local clustering is employed for feature point selection, resulting in enhanced convergence efficiency and improved overall stability of the pigment separation algorithm. Future research will involve further investigations and quantitative evaluations of the separation effectiveness.

Future research will involve further investigations and quantitative evaluations of the separation effectiveness. At present, such quantitative analysis is not conducted in this study due to several practical limitations. Specifically, a reliable quantitative assessment requires establishing a mapping model between image pixel values and actual pigment concentrations. This process demands a large amount of ground-truth data, which involves standardized imaging equipment, controlled acquisition environments, and accurate biochemical or optical measurement systems for melanin and hemoglobin concentrations. Additionally, variations across different skin types, measurement techniques, and quantification protocols must be considered to ensure consistency. These factors collectively pose significant challenges that need to be addressed before a robust quantitative evaluation framework can be implemented.

Although oxygen saturation analysis was not within the scope of this study, the proposed method could theoretically be extended to this domain in future work. Prior studies, such as the hyperspectral imaging system described in [11], have demonstrated the feasibility of quantifying oxygen saturation by acquiring images across multiple wavelengths and applying spectral absorption models to estimate the concentrations of chromophores including melanin, oxyhemoglobin, and deoxyhemoglobin.

In contrast, our method relies solely on standard RGB images, which inherently limits spectral resolution. Nevertheless, two potential avenues may allow for estimating oxygen saturation from RGB data. The first involves further decomposing the hemoglobin component obtained by our method into its oxygenated and deoxygenated forms. This would likely require either fixed separation coefficients, estimated empirically, or the use of synthetic spectral data to model the relationship between hemoglobin states and oxygen saturation.

The second approach would be data-driven: by collecting a large dataset of facial images annotated with corresponding oxygen saturation levels (e.g., from pulse oximetry), a supervised learning model could be trained to predict spatial oxygen saturation maps. These strategies, while challenging, represent promising directions for expanding the clinical applicability of our method in the future.

Although metrics such as SSIM, PSNR, or inter-class variance are commonly used in image quality assessment or segmentation tasks, they are not applicable in our scenario due to the absence of ground truth pigment maps. Unlike synthetic images or standard segmentation benchmarks, skin pigment distributions vary significantly across individuals and cannot be objectively measured without invasive biochemical or optical quantification. Therefore, commonly used internal metrics (e.g., SSIM, PSNR) that require a reference image are not meaningful in this context. Similarly, inter-class variance assumes well-defined spatial regions, which does not align with the continuous nature of pigment distributions.

As explained earlier, a reliable quantitative evaluation of pigment separation would require a comprehensive ground-truth dataset with pixel-level melanin and hemoglobin concentrations, which is currently impractical to obtain. We acknowledge this limitation and plan to explore alternative evaluation strategies in future work, potentially through synthetic data or collaborative clinical imaging studies.

In this study, we selected DL-ICA and related ICA-based methods as baselines due to their relevance to the blind source separation formulation of pigment decomposition. While deep learning approaches such as GANs and U-Net variants have shown strong performance in tasks like skin lesion segmentation and facial image enhancement, they typically require large-scale annotated datasets and are focused on high-level semantic features rather than low-level pigment separation. Moreover, the lack of pixel-level ground truth for melanin and hemoglobin concentrations currently limits the feasibility of supervised deep models in this domain. We acknowledge this as a promising future direction and plan to explore hybrid approaches that combine data-driven learning with physical priors in future work.

## Supporting information

S1 Fig Hemoglobin and melanin images obtained from our method in rectangular skin regions: eyebrow, eye and nose. Reprinted with permission from Shanghai Siyan Software Technology Co., Ltd. under a CC BY license.
(DOCX)

S1 File Content permission form. (PDF)
