# Supplementary material for: A novel skin pigment separation method based on sub-block selection and local clustering
Source: PLoS One. 2025 Oct 14;20(10):e0332849. doi: 10.1371/journal.pone.0332849 (PMC12520397; doi:10.1371/journal.pone.0332849)
Supplement: S1 Fig — Reprinted with permission from Shanghai Siyan Software Technology Co., Ltd. under a CC BY license. (DOCX) [file pone.0332849.s001.docx]

Fig S1 shows the hemoglobin and melanin separation results obtained from our method when applied to rectangular skin regions located on the eyebrow, eye, and nose, respectively. Compared to typical facial skin regions such as the forehead or cheeks, these areas exhibit complex visual characteristics that can negatively impact decomposition performance. Specifically, the eyebrow region contains dense hair structures, the eye region includes curved surfaces and high specular reflections, while the nose region often presents strong highlights and shadows due to its pronounced 3D shape. As a result, the pigment separation results in these areas tend to be less stable or less interpretable. These observations support our decision to focus on more homogeneous skin regions (e.g., forehead) for initial algorithm evaluation and performance benchmarking.


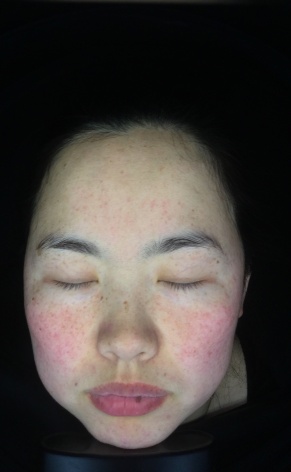


Original image


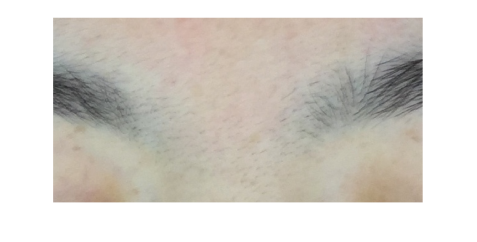


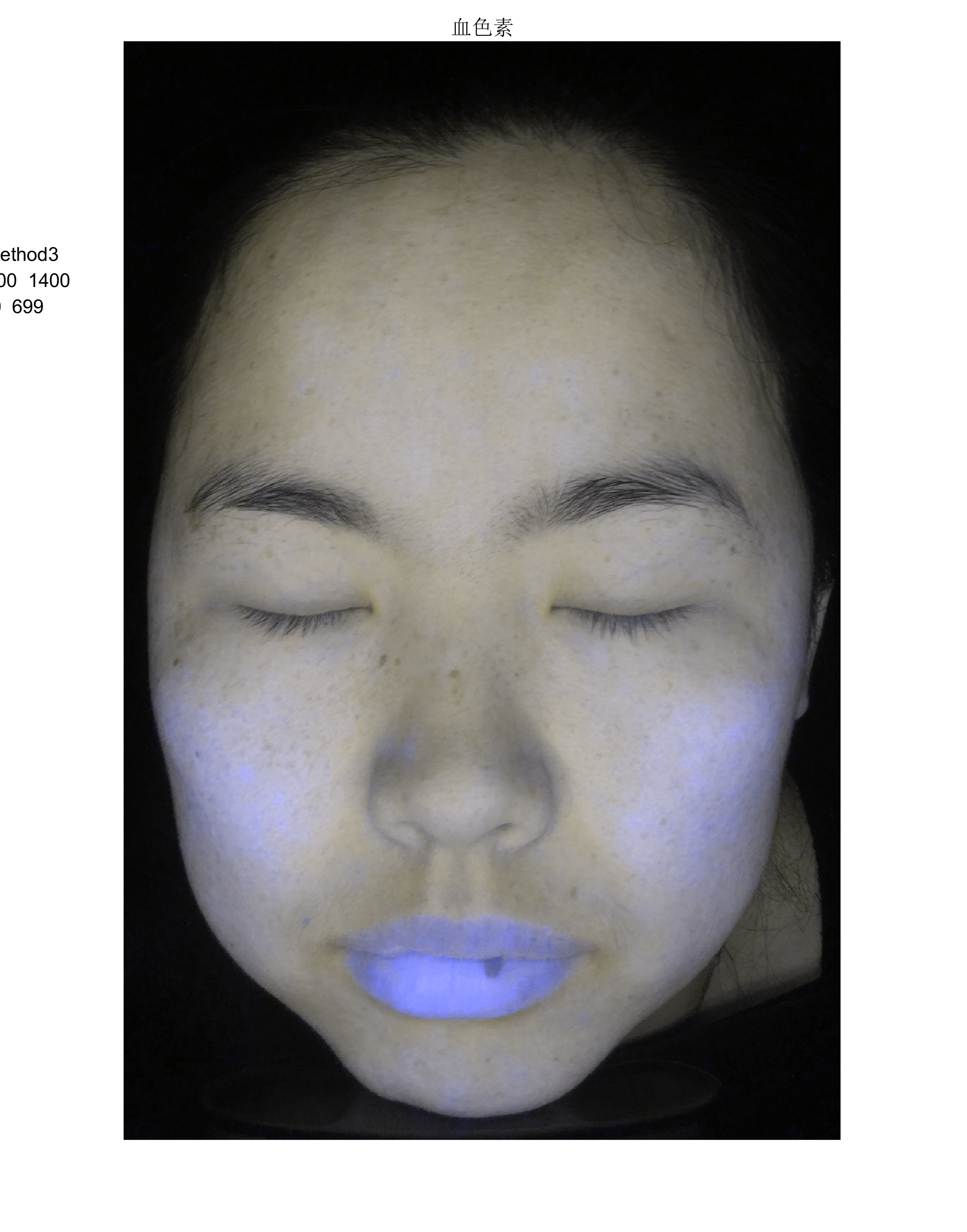

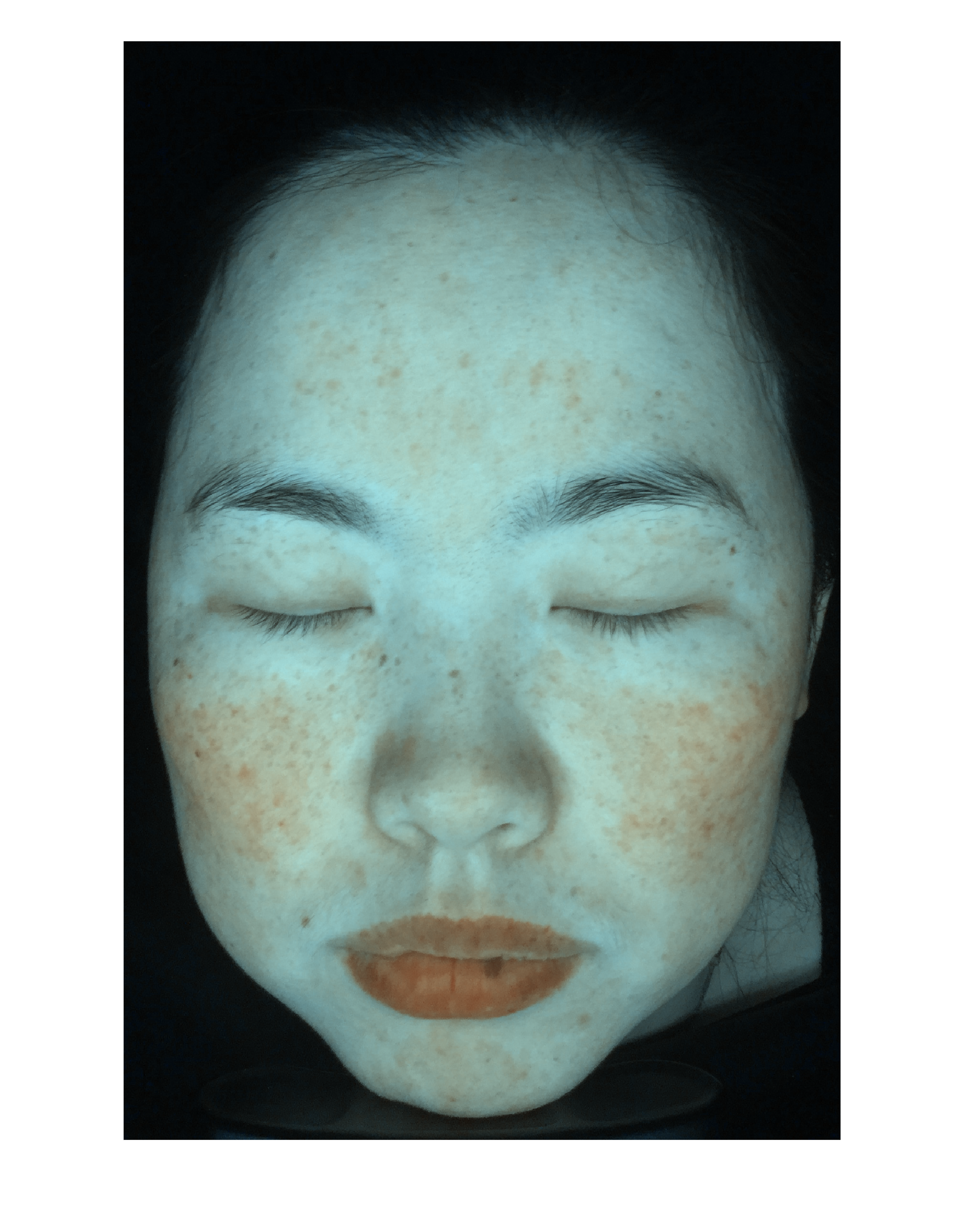


Rectangular skin area on eyebrow Hemoglobin image Melanin image


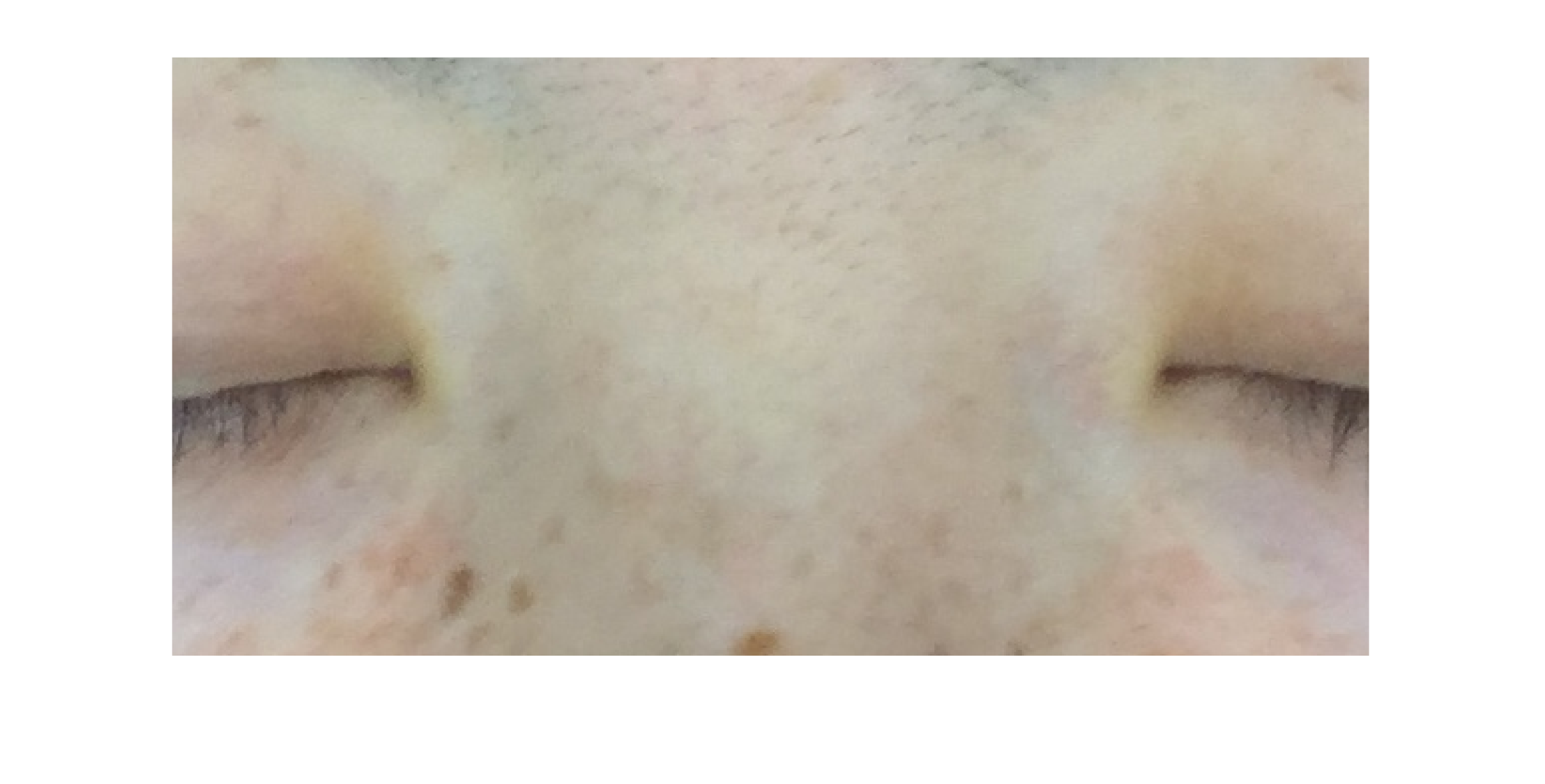

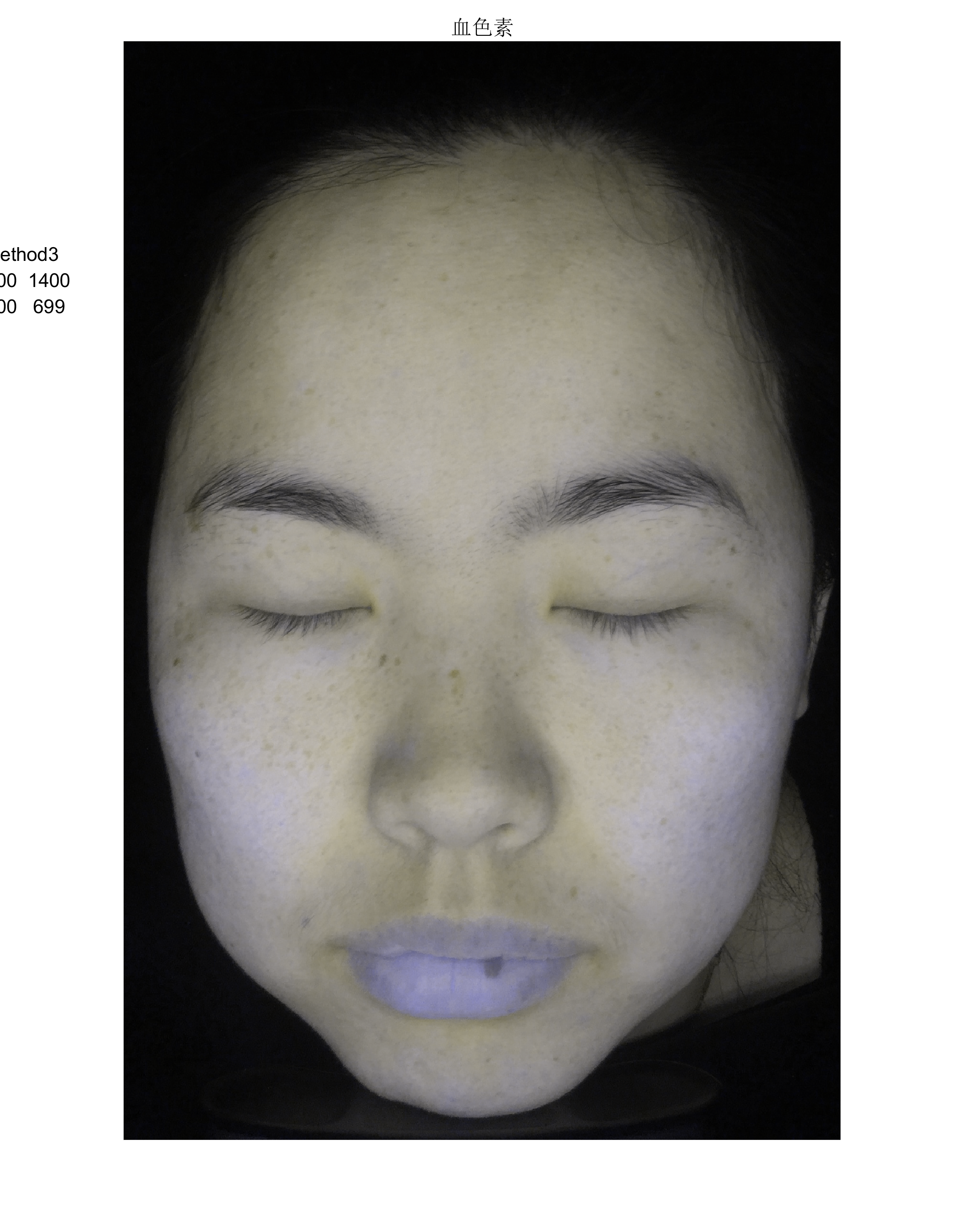

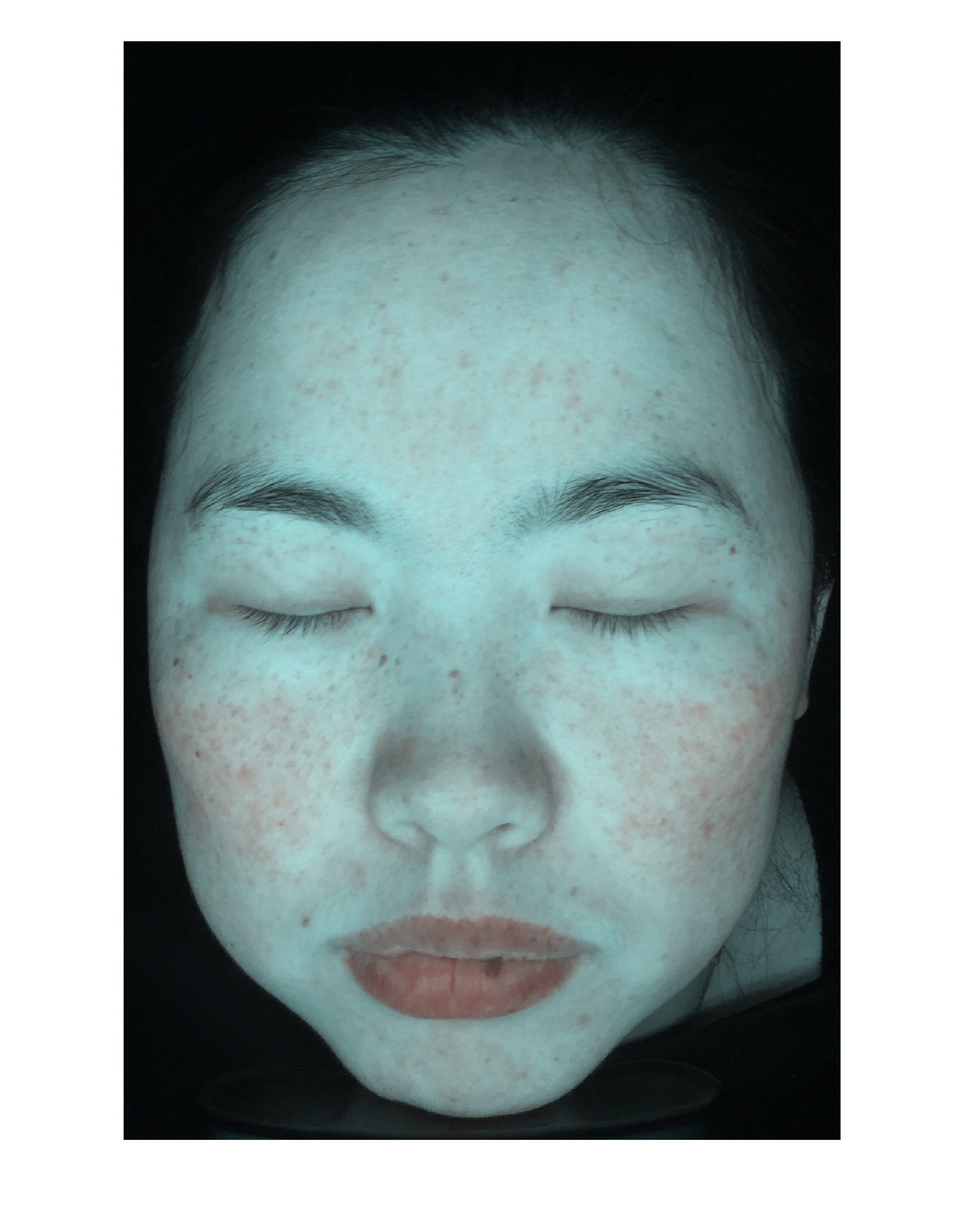


Rectangular skin area on eye Hemoglobin image Melanin image


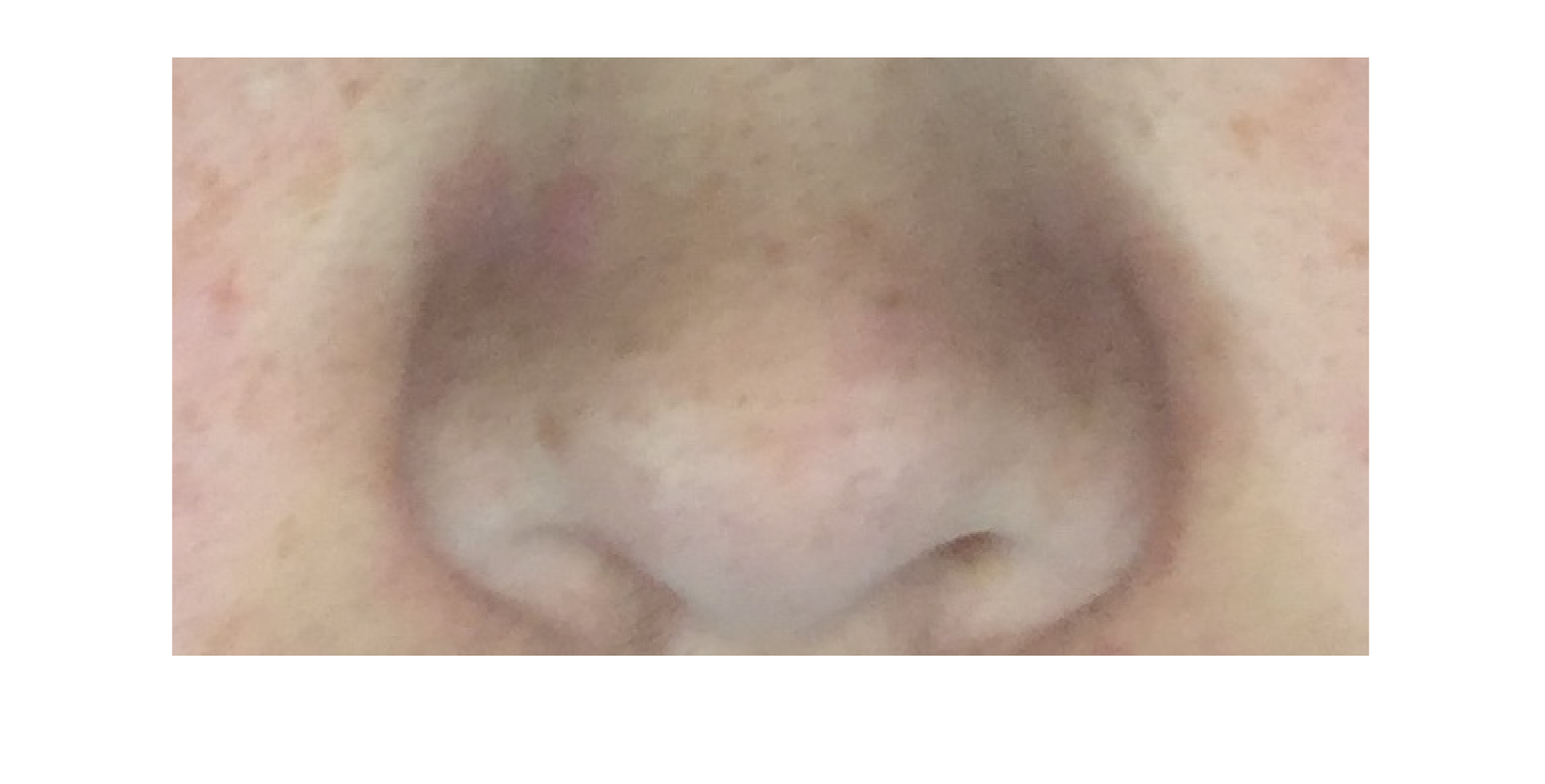

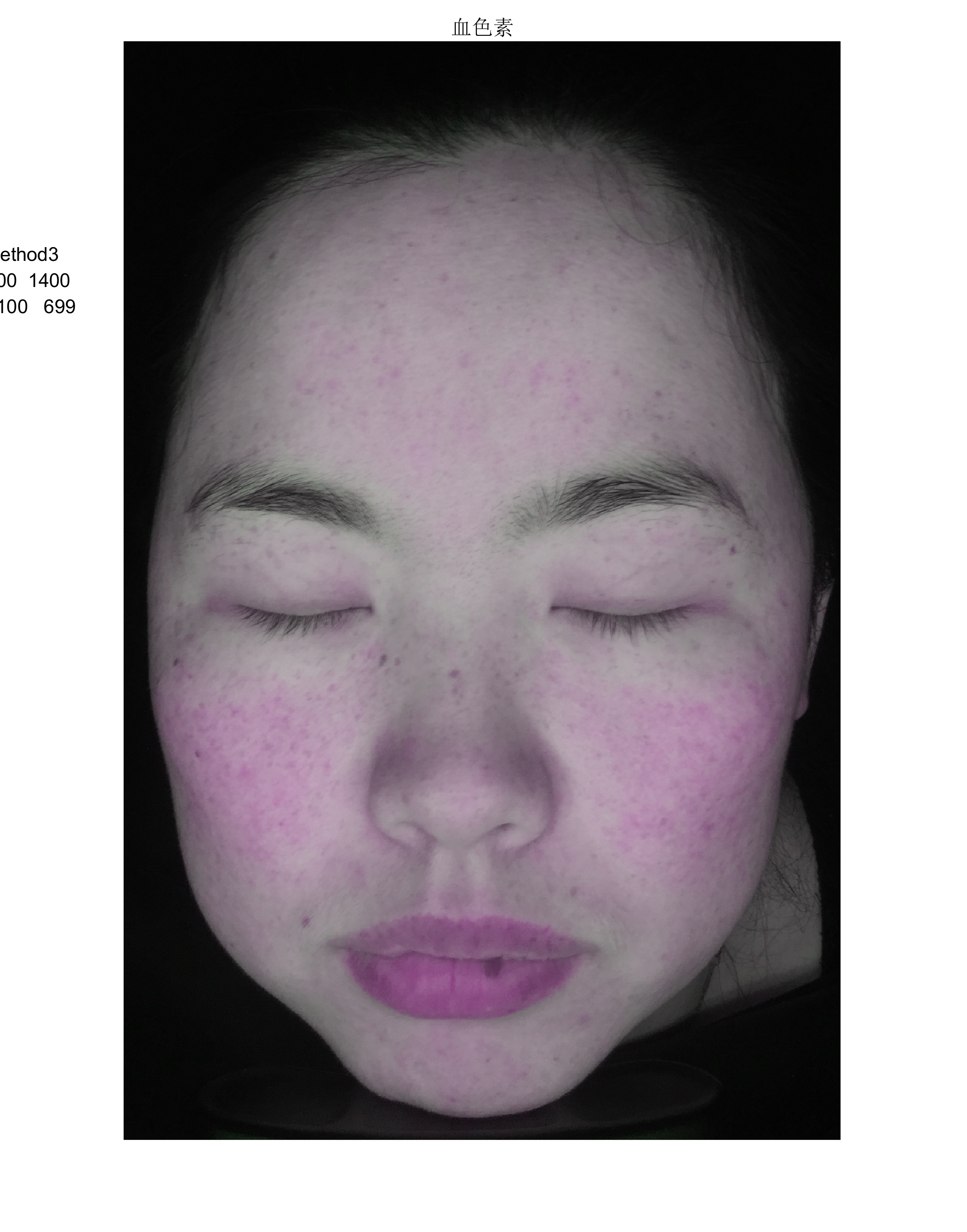

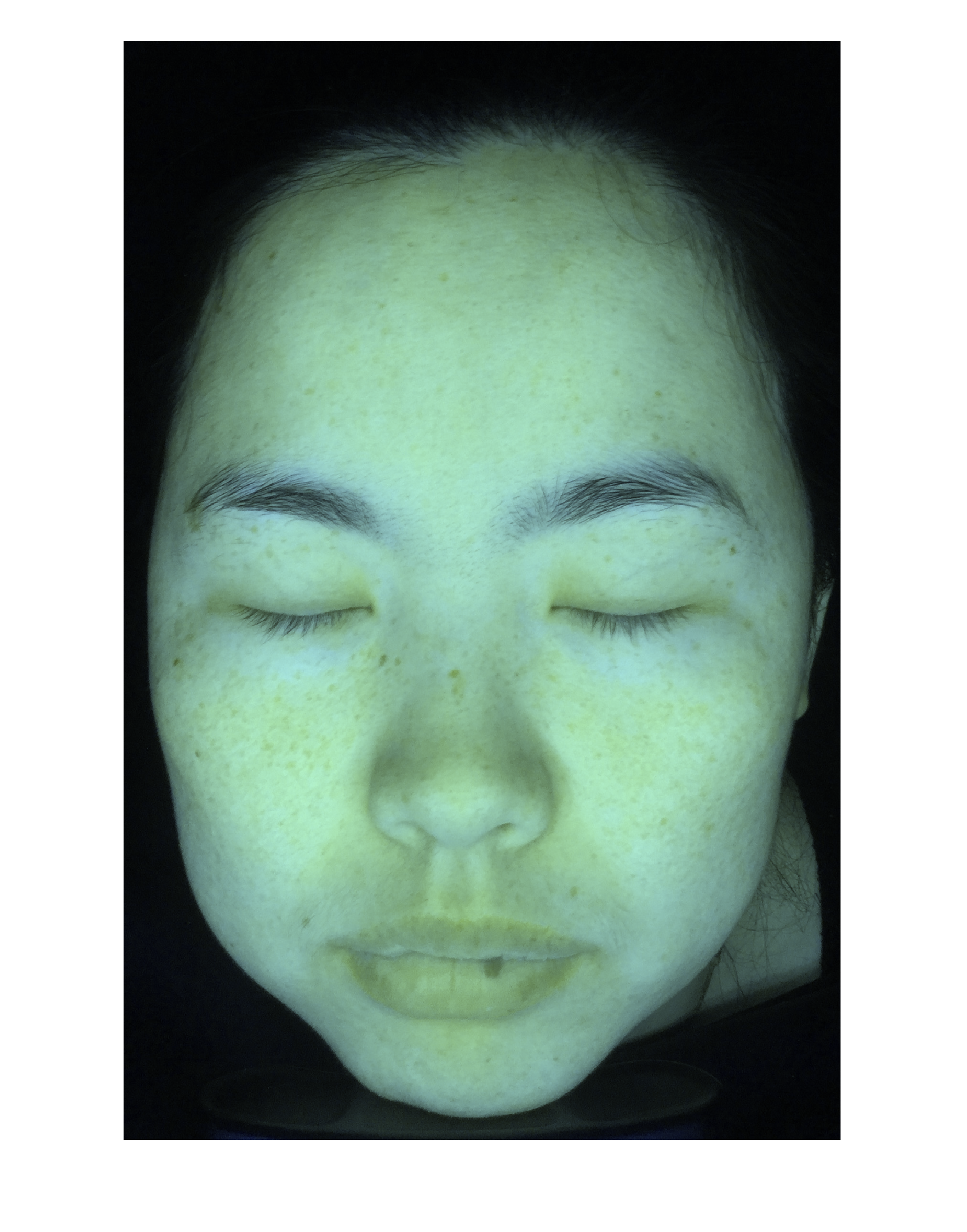


Rectangular skin area on nose Hemoglobin image Melanin image

**Fig S1 Hemoglobin and melanin images obtained from our method in rectangular skin regions: eyebrow, eye and nose**
